# VOLT: a novel open-source pipeline for automatic segmentation of endolymphatic space in inner ear MRI

**DOI:** 10.1007/s00415-020-10062-8

**Published:** 2020-07-14

**Authors:** J. Gerb, S. A. Ahmadi, E. Kierig, B. Ertl-Wagner, M. Dieterich, V. Kirsch

**Affiliations:** 1 Department of Neurology, University Hospital, Ludwig-Maximilians-Universität München, Marchioninistraße 15, 81377 Munich, Germany; 2 German Center for Vertigo and Balance Disorders – IFB-LMU, University Hospital, Ludwig-Maximilians-Universität München, Munich, Germany; 3 Graduate School of Systemic Neuroscience (GSN), Ludwig-Maximilians-Universität München, Munich, Germany; 4 Department of Radiology, University Hospital, Ludwig-Maximilians-Universität München, Munich, Germany; 5 Department of Radiology, The Hospital for Sick Children, University of Toronto, Toronto, Canada; 6 Munich Cluster for Systems Neurology (SyNergy), Munich, Germany

**Keywords:** Endolymphatic hydrops, Inner ear, MRI, Intravenous application, Contrast agent, Volumetric, Local thresholding, Automatic segmentation

## Abstract

**Background:**

Objective and volumetric quantification is a necessary step in the assessment and comparison of endolymphatic hydrops (ELH) results. Here, we introduce a novel tool for automatic volumetric segmentation of the endolymphatic space (ELS) for ELH detection in delayed intravenous gadolinium-enhanced magnetic resonance imaging of inner ear (iMRI) data.

**Methods:**

The core component is a novel algorithm based on Volumetric Local Thresholding (VOLT). The study included three different data sets: a real-world data set (D1) to develop the novel ELH detection algorithm and two validating data sets, one artificial (D2) and one entirely unseen prospective real-world data set (D3). D1 included 210 inner ears of 105 patients (50 male; mean age 50.4 ± 17.1 years), and D3 included 20 inner ears of 10 patients (5 male; mean age 46.8 ± 14.4 years) with episodic vertigo attacks of different etiology. D1 and D3 did not differ significantly concerning age, gender, the grade of ELH, or data quality. As an artificial data set, D2 provided a known ground truth and consisted of an 8-bit cuboid volume using the same voxel-size and grid as real-world data with different sized cylindrical and cuboid-shaped cutouts (signal) whose grayscale values matched the real-world data set D1 (mean 68.7 ± 7.8; range 48.9–92.8). The evaluation included segmentation accuracy using the Sørensen-Dice overlap coefficient and segmentation precision by comparing the volume of the ELS.

**Results:**

VOLT resulted in a high level of performance and accuracy in comparison with the respective gold standard. In the case of the artificial data set, VOLT outperformed the gold standard in higher noise levels. Data processing steps are fully automated and run without further user input in less than 60 s. ELS volume measured by automatic segmentation correlated significantly with the clinical grading of the ELS (*p* < 0.01).

**Conclusion:**

VOLT enables an open-source reproducible, reliable, and automatic volumetric quantification of the inner ears’ fluid space using MR volumetric assessment of endolymphatic hydrops. This tool constitutes an important step towards comparable and systematic big data analyses of the ELS in patients with the frequent syndrome of episodic vertigo attacks. A generic version of our three-dimensional thresholding algorithm has been made available to the scientific community via GitHub as an ImageJ-plugin.

## Introduction

Delayed intravenous gadolinium-enhanced magnetic resonance imaging of the inner ear (iMRI) enables direct, in-vivo, non-invasive verification of endolymphatic hydrops (ELH) simultaneously in both inner ears [1]. This reasonably recent methodical development introduced a broader, more structured investigation to the clinical syndromes associated with ELH, which up to then was thought to be pathognomonic to Menière’s disease (MD) [2]. Today, the relationship between ELH and MD symptoms (for review cp [3]), as well as the specificity of ELH for MD, has come under scrutiny. The underlying reason is that different ELH patterns can be found not only in MD [4, 5], but also so far in 3.3–28% of healthy ears [6, 7], various inner ear [8–10] and central [11–14] pathologies, as well as in anatomic or vascular abnormalities affecting endolymph resorption [15–17].

Because of this, objective and volumetric quantification is considered a necessary step to assess and compare ELH results. So far, the clinical gold standard assessment of the endolymphatic space (ELS) is based on a semi-quantitative and subjective grading reliant on a few MR slices in a transversal plane. Current ELS MR volumetric assessment approaches propose either manual or semi-automatic segmentation [18]. Already a considerable improvement, these approaches lack normalization and require lengthy user interaction that is not suitable for use in more extensive group studies or clinical routine.

Here, we introduce a novel tool for automatic volumetric segmentation of the ELS for ELH detection in iMRI data. The core component is a novel three-dimensional algorithm based on Volumetric Local Thresholding (VOLT). The tool was validated on artificial and prospective real-world data sets.

## Materials and methods

### Data sets

The study included three different data sets: data set 1 (D1, *development data set*) was used to develop the novel ELH detection algorithm based on Volumetric Local Thresholding (VOLT). Data set 2 (D2, artificial validation data set) and data set 3 (D3, prospective validation data set) were used to validate VOLT on entirely unseen data.

D1 and D3 included real-world data sets from consecutive patients from the interdisciplinary German Center for Vertigo and Balance Disorders (DSGZ) of the Munich University Hospital (LMU) between 2015 and 2019. Institutional Review Board approval was obtained before the initiation of the study (no 64115). Included patients had presented with episodic vertigo attacks [19] and undergone iMRI as part of their indicated clinical diagnostic workup to evaluate their ELS. Their data sets were included after they had given oral and written consent following the Declaration of Helsinki. The inclusion criteria were age above 18 years. Exclusion criteria were any MR-related contraindications [20], poor image quality, or missing MR sequences. D1 included 210 inner ears of 105 consecutive patients (50 male; aged 19–84 years, mean age 50.4 ± 17.1 years), and D3 included 20 inner ears of 10 consecutive patients (5 male, aged 31–69 years, mean age: 46.8 ± 14.4 years). D1 and D3 did not differ significantly concerning age, gender, the grade of endolymphatic hydrops (ELH), or data quality (intensity, mean grayscale value). A detailed description of D1and D3 is given in Table 1.

**Table 1:** Description of the real-world data sets

	*N* (gender)	Age	Diagnosis	ELH	ELH grade	Data Quality
D1	105 (50 male)	50.4 ± 17.1 range 19–84	32% VM (*n* = 33) 28% MD (*n*= 29) 18% NV (*n* = 19) 17% VP (*n* = 18) 3% BVP (*n* = 4) 2% BPPV (*n* = 2)	97 out of 210 ears 46.2%	0.7 ± 0.8 Range 0–3	1.1 ± 0.3 Range 0.3–2.3
D3	10 (5 male)	46.8 ± 14.4 range 31–69	10% VM (*n* = 1) 70% MD (*n* = 7) 10% NV (*n* = 1) 10% BPPV (*n* = 1)	7 out of 20 ears 35%	0.7 ± 0.9 Range 0–2.5	1.1 ± 0.3 Range 0.3–1.6

D1 and D3 included data sets from consecutive patients from the interdisciplinary German Center for Vertigo and Balance Disorders (DSGZ), Munich, Germany. Included patients had presented with episodic vertigo attacks and undergone delayed intravenous gadolinium-enhanced magnetic resonance imaging of the inner ear (iMRI) as part of their indicated clinical diagnostic workup. Patients were clinically diagnosed according to the several international guidelines, most of the classification committee of the international Bárány Society (https://www.jvr-web.org/ICVD.html or https://www.baranysociety.nl) and included the diagnosis of VM [23], MD [24], VP [25], BPPV [26], BVP [1] and acute unilateral vestibulopathy/vestibular neuritis [2]. Grading of the ELH in the vestibulum and cochlea was based on criteria described previously [3], which constitutes a fusion of two classification systems [4, 5]. D1 and D3 did not differ significantly concerning age, gender, the grade of ELH, or data quality

± standard deviation, *BPPV* benign paroxysmal positional vertigo, *BVP* bilateral vestibulopathy, *ELH* endolymphatic hydrops, *ELS* endolymphatic space, *iMRI* delayed intravenous gadolinium-enhanced magnetic resonance imaging of the inner ear, *MD* Menière’s disease, *N* number of participants, *VM* vestibular migraine, *VP* vestibular paroxysmia

**Fig. 1:**
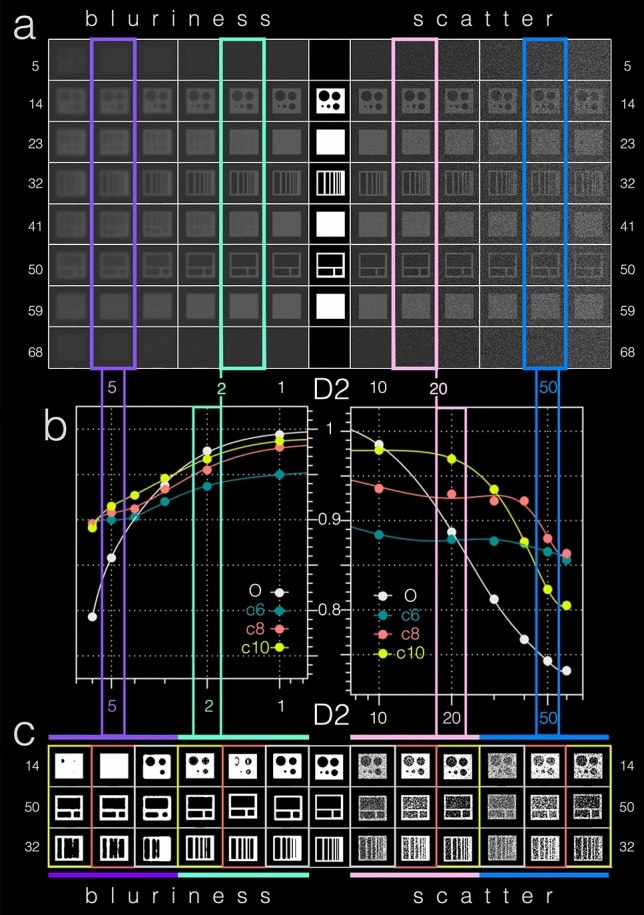
D2 artificial data set–visualization and results. As an artificial data set, D2 provided a known ground truth to test and compare VOLT cutoff versions to Otsu’s method. **a** A transversal slice-wise visualization of D2 in the middle. D2 can be viewed in the very middle and included an 8-bit cuboid volume with different sizes of cylindrical and cuboid-shaped cutouts (signal). To this signal different types of real-world MRI imitating noise were added stepwise in the form of increasing blurriness (Gaussian blur kernel, SD range 1–6 voxel in x/y/z-direction; SD = standard deviations, visualized to the left) and increasing scatter (SD range of intensity variation: 0–50 SD, visualized to the right). **b** Based on empirical observations in the development data set (D1), VOLT was compared to Otsu’s method (O = grey) at three cutoff variations (c6 = forest green, c8 = red, c10 = yellow). Both VOLT cutoff versions and Otsu’s method fared better with blurriness noise (*x*-axis of the left graph) in comparison with scatter noise (*x*-axis of the right graph). More specifically, VOLT cutoff versions showed a high level of agreement in terms of Dice overlap (*y*-axis within the graphs) with Otsu’s scores in data sets with low noise levels (please compare blurriness 2, framed in mint green and scatter 20, framed in pink). The higher the noise level, the more VOLT cutoff versions outperformed Otsu’s method (please note blurriness 5, framed in purple and scatter 50, framed in blue). The corresponding output (**c**) can easily be compared with the ground-truth by following said color frames. *D*2 data set 2, *c*6 cutoff 6, *c*8 cutoff 8, *c*10 cutoff 10, *O* Otsu’s method

As an artificial data set, D2 provided a known ground truth to test and compare VOLT’s performance to an adapted version of Otsu’s method [21], which is a recognized foreground/background segmentation algorithm based on global thresholding at an optimal histogram-derived cutoff. D2 consisted of an 8-bit cuboid volume using the same voxel-size and grid as real-world data with different sized cylindrical and cuboid-shaped cutouts (signal) whose grayscale values matched the real-world data set D1 (mean 68.7 ± 7.8; range 48.9–92.8). To this structural basis signal, two types of noise were added, which imitate the real-world variability of MRI signals [22]. The noise was added stepwise in the form of increasing blurriness noise (Gaussian blur kernel, SD range 1–6 voxel in x/y/z-direction; SD = standard deviations) or increasing scatter noise (Gaussian, SD range of intensity variation: 0–50 SD). D2 and its varying levels of noise can be viewed in Fig. 1a.

### D1 and D3–Clinical diagnosis and measurement of the auditory, semicircular canal, and otolith functions

Patients were clinically diagnosed according to the international guidelines, most of the classification committee of the international Bárány Society (www.jvr-web.org/ICVD.html or www.baranysociety.nl) for the diagnosis of vestibular migraine [23], Menière’s disease [24], vestibular paroxysmia [25], bilateral vestibulopathy [26], acute unilateral vestibulopathy/vestibular neuritis [27] and benign paroxysmal positional vertigo [28]. The diagnoses of the patients within D1 and D3 can be viewed in Table 1.

Diagnostic workup included a careful neurological and neuro-otological examination including neuro-orthoptic assessment (e.g., Frenzel goggles; fundus photography and adjustments of the subjective visual vertical (SVV) for graviceptive vestibular function, for methods, see [29]), video-oculography during the head-impulse test (vHIT) for dynamic vestibular function (for methods, see [30, 31]), audiometry, and MR imaging of the whole brain including the cerebellopontine angle and brainstem.

### D1 and D3–Sequence protocol and grading of the delayed gadolinium-enhanced ivMRI of the inner ear

Four hours after intravenous injection of a standard dose (0.1 ml/kg body weight, i.e., 0.1 mmol/kg body weight) of Gadobutrol (Gadovist®, Bayer, Leverkusen, Germany), MR imaging data were acquired in a whole-body 3 T MR scanner (Magnetom Skyra, Siemens Healthcare, Erlangen, Germany) with a 20-channel head coil. Head movements were minimalized in all three axes using a head positioning system for MRI (Crania Adult 01, Pearl Technology AG, Schlieren, Switzerland). A 3D-FLAIR sequence was used to differentiate endolymph from perilymph and bone, and a CISS sequence to delineate the total inner ear fluid space from the surrounding bone. A T2-weighted, three-dimensional, fluid-attenuated inversion recovery sequence (3D-FLAIR) with the following parameters: TR 6000 ms, TE 134 ms, TI 2240 ms, FOV 160 × 160 mm^2^, 36 slices, base resolution 320, averages 1, acceleration factor of 2 using a parallel imaging technique with a generalized auto-calibrating partially parallel acquisition (GRAPPA) algorithm, slice thickness 0.5 mm, acquisition time 15:08 min was carried out. A high-resolution, strongly T2-weighted, 3D constructive interference steady state (CISS) sequence of the temporal bones was performed to evaluate the anatomy of the whole-fluid-filled labyrinthine spaces with the following parameters: TR 1000 ms, TE 133 ms, FA 100°, FOV 192 × 192 mm^2^, 56 slices, base resolution 384, averages 4, acceleration factor of 2 using GRAPPA algorithm, slice thickness of 0.5 mm and acquisition time 8:36 min. The presence of ELH was observed on the 3D-FLAIR images as enlarged negative-signal spaces inside the labyrinth, according to a previously reported method [32, 33]. The decision to apply a single-dose contrast agent was made because of the ongoing discussion about gadolinium deposition within the dentate nucleus and globus pallidus after repeated administration of gadolinium-based contrast agents [34–37]. It was not considered ethical to apply higher doses of contrast agent if not necessary. Accordingly, only patients with a diagnostic benefit were included in the study.

Evaluation of the iMRI and grading of the ELS was performed independently by two experienced head and neck radiologists and a neurologist who was blinded to the clinical patient data. If discrepancies arose, a consensus was reached by discussion. The characterization of the ELS in the vestibulum and cochlea was based on criteria previously described [12], which constitutes a fusion of two classification systems [38, 39]. D1 and D3 did not differ significantly concerning the grade of ELH. An overview of ELH grade and data quality for data sets D1 and D3 can be viewed in Table 1.

### D1–Development of the automatic segmentation tool for ELH detection based on Volumetric Local Thresholding (VOLT)

**Fig. 2:**
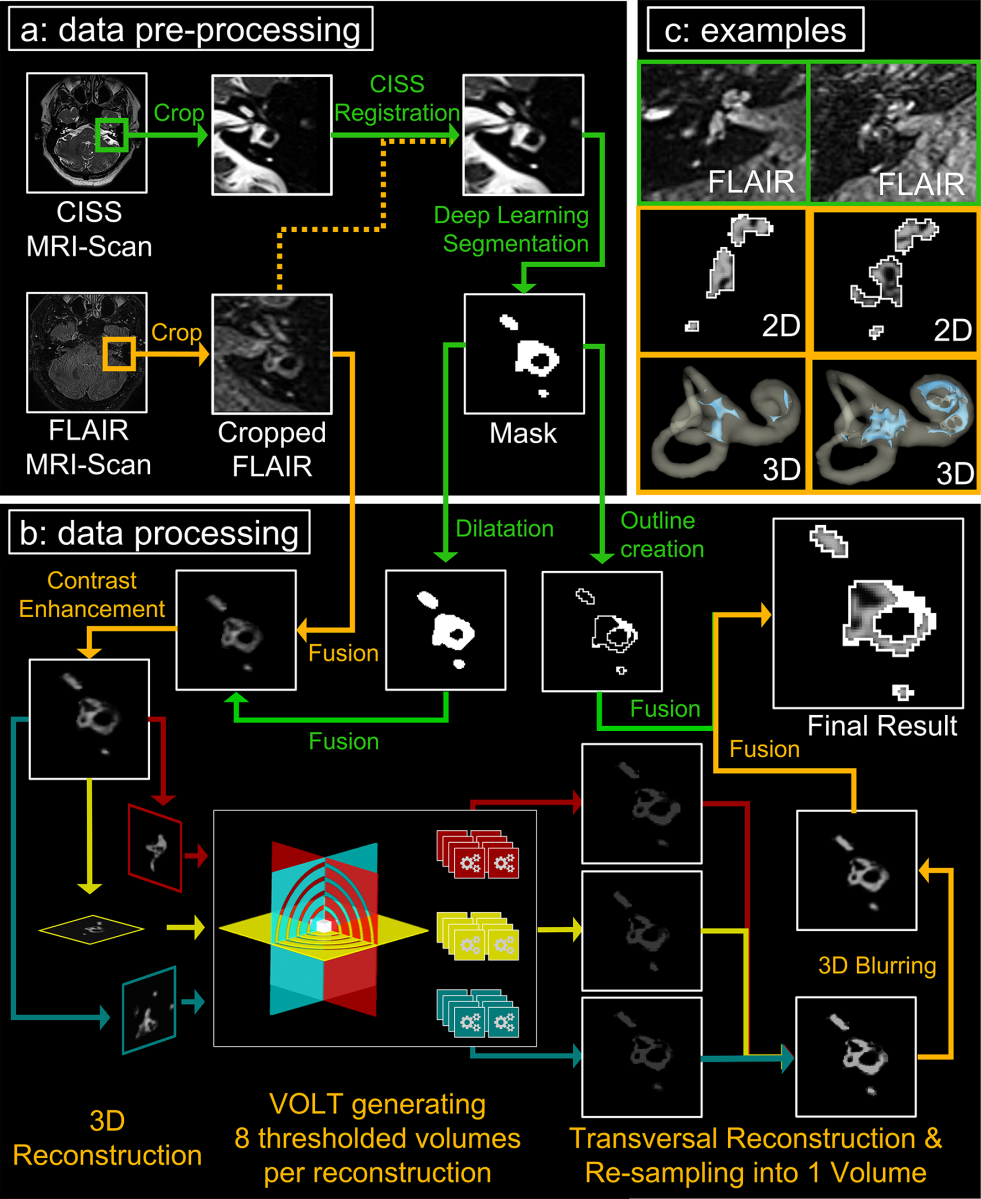
VOLT flowchart and output examples. The flowchart shows a step-by-step overview of the VOLT processing pipeline of a left inner ear. The different steps correspond to the boxes in a counterclockwise fashion (a, b, c). **a** Describes data pre-processing, **b** data processing, and **c** shows output examples. Within each box, processing steps following orange arrows indicate the order of the main program steps, and green arrows indicate supporting steps. Data pre-processing (a) consists of cropping the inner ear from CISS and FLAIR MR images (only step requiring user input), co-registration, and using a cloud-based deep convolutional neural network (CNN) to create a mask of the inner ear. During data processing, (b) the mask is dilated to include a small seam around the inner ear region-of-interest (ROI). Then, a fusion volume is created, contrast-enhanced, and the fusion volume is 3D reconstructed. VOLT is performed, volumes are reconstructed into a transversal plane and re-sampled into one volume. After 3D blurring, single-voxel noise is removed, and a three-dimensional outline based on the mask is added to the final result. (c) depicts two output examples of the right inner ear. The upper row shows the corresponding cropped FLAIR-MR image; the middle row shows a 2D depiction of the VOLT output, and the lower row shows the 3D visualization of VOLT-output. The inner ear to the left displays no endolymphatic hydrops (ELH). The inner ear to the right displays an ELH grade 2. *CISS* constructive interference in steady-state, *MR* magnetic resonance, *FLAIR* fluid-attenuated inversion recovery, *VOLT* volumetric local thresholding

VOLT was developed on the real-world data set D1 using exclusively universal access software, namely 3D Slicer version 4.11 toolbox [40] including the TOMAAT plugin [15], as well as ImageJ Fiji [41] including the “Fuzzy and artificial neural networks image processing toolbox” [42] and the “MorphoLibJ Toolbox” [43](see an overview of the overall pipeline including VOLT-based ELS segmentation in Fig. 2a, b).

#### Data pre-processing included the following steps

VOLT operates on a pre-segmented region-of-interest (ROI) of the inner ear, which requires a series of data pre-processing steps. First, FLAIR and CISS sequences were interpolated to a voxel size of 0.25 mm × 0.25 mm × 0.25 mm using a bicubic interpolation algorithm in ImageJ. Then, left and right inner ears were cropped using a rectangular selection, converted to nrrd-files, and imported into 3D-Slicer. To obtain an ROI of the overall inner ear region, a voxel-wise segmentation of this cropped region needed to be obtained. To this end, we applied a recently proposed deep convolutional neural network (CNN), deployed via the TOMAAT module in 3D–Slicer [44]. This step first normalizes the orientation of the cropped volume by affine image registration (BRAINSFit toolkit [45]) and then applies a pre-trained volumetric CNN with V-net architecture [46]. The V-net output yields a segmentation into two labels, either inner ear or background. The “inner-ear” segmentation, hereafter referred to as “mask”, was converted into an 8-bit binary volume and volumetrically dilated using 3D morphological filtering. As dilation adds a thin shell of anatomy surrounding the inner ear, this step allows the amount of false-negative classifications by the VOLT segmentation algorithm to be reduced. An overview of the pre-processing required for VOLT-based ELS segmentation can be viewed in Fig. 2a.

#### Data processing included the following steps

Two locally adaptive thresholding algorithms (“Bernsen” [47] and “Mean”) were used in the three planes, and in four varying radii, respectively, to differentiate between endolymphatic fluid (EF) and non–endolymphatic fluid (NEF). These intermediate segmentations were then reconstructed in a transversal plane and aggregated into one final segmentation volume. Close attention was paid to avoid the inclusion of false positives into the ELS, by considering only voxels within a volumetrically strict outer shell of the inner ear (see pre-processing). As a next step, single-voxel-noise was reduced using a 3D Gaussian blurring algorithm. The first mask was used to create a single pixel-sized borderline (= 0.25 mm) in all three planes to ultimately avoid false-positive classifications in the corner regions of these three planes. The resulting 3D volume can then be regarded as a probabilistic map of the inner ear, which included the classification into its two different compartments (endolymphatic and perilymphatic space). The final classification then strongly depends on the chosen cutoff. Each cutoff matches a percentage of positive classifications. For example, cutoff 6 (c6) corresponds to 79.2%, cutoff 8 (c8) to 70.8% and cutoff 10 (c10) to 62.5% classifications into endolymphatic space. Based on empirical observations in the development data set (D1), VOLT was validated at three cutoff variations (c6, c8, c10; Fig. 2b).

#### Automatization and pipeline creation

A script written in the IJ1M-macro-language was used to automate pre-processing and processing in FIJI. User input was required solely for supervision purposes during cropping, registration, and segmentation. The remaining features (pre-processing, volumetric reconstruction, contrast enhancement, fusion, thresholding, and post-processing) work automatically.

### D1, D2 and D3–Methods for validation

VOLT with three different cutoffs (c6, c8, c10) was validated on the artificial data set D2 and the prospective real-world data set D3. Segmentation accuracy was evaluated using the Sørensen-Dice overlap coefficient, which is defined as 2 * |X ∩ Y|/|X| +|Y| for segmentations X and Y [48], as a measure of region overlap between gold standard segmentation and the automatically obtained segmentations from the VOLT pipeline.

Segmentation precision was estimated by comparing the volume of the ELS (V_E_) between segmentation methods. Structurally, the human inner ear can be pictured as an external, bony hose system (called the bony labyrinth, containing perilymph) and an inner hose system (called the membranous labyrinth, containing endolymph). The total lymph fluid space includes the inner hose system’s ELS and the surrounding perilymphatic space.

Receiver operating characteristics (ROC) analysis was used to show the (in)dependence of the performance of the methods from the grade of the ELH or the distribution of the fluids within the total fluid space (TFL) and the SNR of the iMRI data set.

### D1, D2 and D3 statistics and map display

The data were analyzed with SPSS 20.0 (SPSS, Chicago, IL, USA). Differences between data sets overall were assessed using a paired t-test, which was Bonferroni-corrected for multiple testing and viewed at *p* < 0.01 and *p* < 0.05. Linear agreement between parameter pairs was calculated for each method separately using the two-sided Spearman’s correlation coefficient and reported at a significance level of *p* < 0.01 and *p* < 0.05. For Receiver operating characteristics (ROC) analysis, the original Fortran program JLABROC4 (by Charles Metz and colleagues, Department of Radiology, University of Chicago; Java translation by John Eng, Russel H Morgan Department of Radiology and Radiological Science, Johns Hopkins University, Baltimore, Maryland, USA, Version 2.0, March 2017) was used.

## Results

### VOLT implementation on D1

After implementation on data set D1, the novel tool for automatic segmentation of the endolymphatic space (ELS) with a novel algorithm based on Volumetric Local Thresholding (VOLT) ran smoothly and showed no operational or stability issues. VOLT does not require especially powerful hardware or closed-source software. The only prerequisite is the installation of universal access software, namely 3D Slicer toolbox [40] including the TOMAAT plugin [15], as well as ImageJ Fiji [41] including the “Fuzzy and artificial neural networks image processing toolbox” [42] and the “MorphoLibJ Toolbox” [43].

The only step requiring user input was the cropping step. While this step required a rough selection of the inner ear and could easily be automatized, it allowed a quick and easy visual assessment of the source images and was therefore considered a suitable quality control mechanism. Cropping was performed in order to reduce computation time as well as allow for easier registration of the inner ears; the registration step was necessary to ensure correct positioning of the CISS-based hull relative to the FLAIR. For both registration and CNN segmentation, the necessary user input was limited to entering parameters and starting the process. After the CNN segmentation the user had to save the segmentations as a new file manually. As an orientation, pre-processing steps of one single-subject data set can be performed in less than ten minutes by an experienced user on a standard consumer laptop (Windows10 (64Bit), Intel^®^ Core i5-4200U @1,6 GHz, 8 GB RAM).

Data processing steps are fully automated and run without further user input in less than 60 s. Volumetric local thresholds can be adapted to signal-to-noise ratio (SNR) of different data sets. Output files include 3D volumetric quantification of TLS and ELS in mm^3^ and a 3D visualization of the inner ear. Examples of single-subject VOLT-based inner ear segmentations show different grades of ELH (Fig. 2c).

### VOLT performance on artificial data set D2

D2 was created to have a ground truth data set featuring challenges found in inner ear imaging, namely low contrast and high noise. Similar to actual iMRI, the regions of interest were three-dimensional volumes of different sizes. This proofed to be difficult for 2D-algorithms, whereas three-dimensional methods could analyze the data set better.

As an artificial data set, D2 provided a known ground truth to test and compare VOLT’s performance to an adapted version of Otsu’s method (O) [21], which is a recognized foreground/background segmentation algorithm based on global thresholding at an optimal histogram-derived cutoff. Based on empirical observations in the development data set (D1), VOLT was compared to O at three cutoff variations (c6, c8, c10). On average, over all noise conditions, the Dice score (DS) of VOLT cutoff versions (c6: 90%, c8: 92%; c10: 92%) outperformed Otsu’s method (82%). Both VOLT cutoff versions and Otsu’s method fared better with blurriness noise (DS:O: 91%; c6: 92%, c8: 93%; c10: 94%) in comparison with scatter noise (DS O: 82%; c6: 87%, c8: 91%; c10: 90%). More specifically, VOLT cutoff versions showed a high level of agreement in terms of Dice overlap with Otsu’s scores in data sets with low noise levels (Bl 1–4; Sc 10). The higher the noise level, however, the more VOLT cutoff versions outperformed Otsu’s method (Bl 5–6; Sc 20–60), with c8 showing an overall best performance independent of noise levels. All results are presented in Table 2 and Fig. 1b, c.

**Table 2:** Overview of results

A						
Data set	Noise	Scale	Otsu’s	Cutoff 6	Cutoff 8	Cutoff 10
D2	BI	1	99.4%	95.0%	98.0%	98.7%
		2	97.6%	93.7%	95.5%	96.7%
		3	93.8%	92.0%	93.4%	94.6%
		4	90.3%	90.3%	91.2%	92.7%
		6	85.8%	90.0%	90.8%	91.5%
	Sc	10	98.5%	88.4%	93.6%	97.9%
		20	88.7%	87.9%	93.0%	96.9%
		30	81.2%	87.7%	92.2%	93.5%
		40	76.7%	87.4%	90.5%	87.6%
		50	74.3%	86.5%	88.0%	82.3%
		60	73.2%	85.6%	86.3%	80.5%

B					
Data set	Validation	M	Cutoff 6	Cutoff 8	Cutoff 10
D3	DS	Gold standard	97.0% ± 0.7 range 95.6–97.9	96.6% ± 0.8 range 95.0–97.7	95.9% ± 0.9 range 93.8–97.2
	V_E_	16.7 mm^3^ ± 5.5 range 8.8–30.7	11.5 mm^3^ ± 5.7 range 5.0–25.5	17.1 mm^3^ ± 7.4 range 8.4–33.6	23.3 mm^3^ ± 8.7 range: 13.0–41.0
	V_E_/M	1	0.7 ± 0.2 range 0.4–0.9	1 ± 0.2 range 0.7–1.5	1.4 ± 0.3 range 1.0–2.1
	V_T_	276.2 mm^3^ ± 37.6 (range 223.6–347.6)			

As an artificial data set, D2 provided a known ground truth to test and compare VOLT cutoff versions to Otsu’s method (O). **A** shows an overview of the Dice scores (DS) of each segmentation method (Otsu’s, cutoff 6, cutoff 8, cutoff 10) concerning the real-world MRI imitating noise that was added stepwise in the form of increasing blurriness noise (Bl, Gaussian blur kernel, SD range 1–6 voxel in x/y/z-direction; SD = standard deviations) or increasing scatter noise (Sc, SD range of intensity variation: 0–50 SD). For visualization of the added noise and results, see Fig. 1a. D3 included real-world data sets from consecutive patients from the interdisciplinary German Center for Vertigo and Balance Disorders, Munich, Germany. Part **B** shows an overview of the results’ mean of each segmentation method (manual segmentation that was considered as the gold standard and VOLT with three different cutoffs 6, 8, 10). Segmentation accuracy was evaluated using the Sørensen-Dice overlap coefficient, and segmentation precision were estimated by comparing the volume of the ELS (V_E_). The ratio V_E_/M was supplied to show the deviation of each cutoff from the gold standard, which was the manual segmentation. The V_E_ ranges include all different grades of endolymphatic hydrops

± standard deviation, *Bl* blurriness, *DS* Dice score, *Sc* scatter, *V*_*E*_ volume of the endolymphatic space, *V*_*T*_ volume of the total fluid space

### VOLT performance on prospective real-world data set D3

**Fig. 3:**
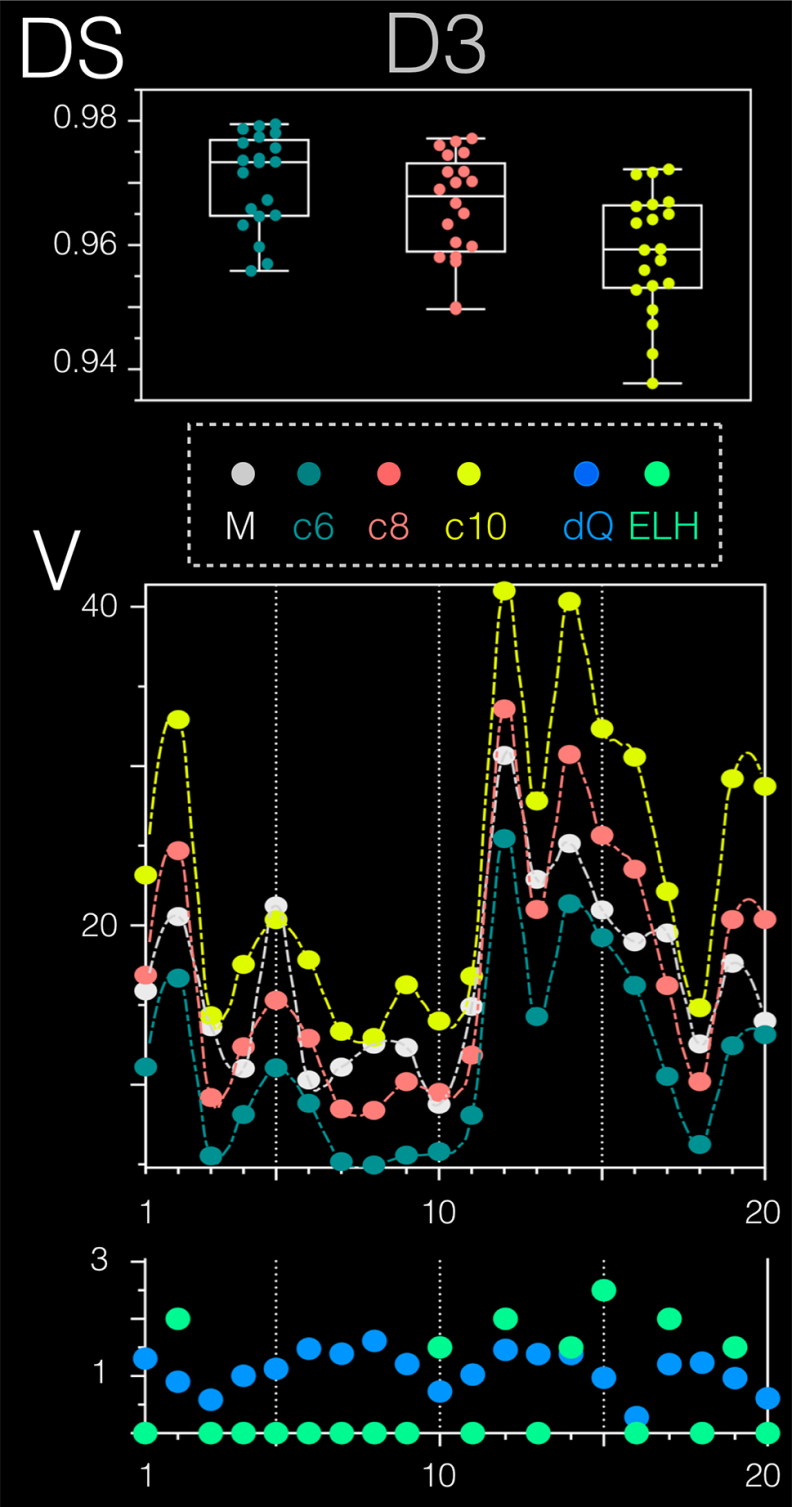
D3 prospective validation data set results. D3 was used to validate VOLT on entirely unseen real-world data (20 inner ears). VOLT with the three variations cutoff 6 (c6 = dark green), cutoff 8 (c8 = red), and cutoff 10 (c10 = yellow) were compared to manual (M) segmentation (= grey, that was considered the gold standard). Ear-specific segmentation accuracy was evaluated using the Sørensen-Dice overlap coefficient (DS, upper graph), and segmentation precision were estimated by comparing the volume of the ELS (V, middle graph). Overall, DS of all three VOLT variations was high (c6: 97.0%$\pm$ 0.7, c8: 96.6%$\pm$ 0.8, c10: 95.9% 97%$\pm$ 0.9). The influence of endolymphatic hydrops (ELH = colored light green) and data quality (dQ = colored blue) can easily be seen in the lowest graph. Data quality was defined as mean the greyscale value (or intensity). Note that the grade of ELH correlated significantly with the endolymphatic volume of both the manual segmentation method (*p* < 0.05) and VOLT cutoff variations c6-8–10 (*p* < 0.01). *c*6 cutoff 6, *c*8 cutoff 8, *c*10 cutoff 10, *D*3 data set 3, *dQ* data quality, *DS* Dice score, *M* manual segmentation

D3 included previously entirely unseen real-world data sets from 10 consecutive patients (= 20 inner ears) and was used to validate VOLT on entirely unseen data. Ear-specific segmentation accuracy was evaluated using the Sørensen-Dice overlap coefficient (DS), and segmentation precision were estimated by comparing the volume of the ELS (V_E_). Performance (DS) of VOLT with the three different cutoffs c6: 97.0% ± 0.7, c8: 96.6% ± 0.8, c10: 95.9% 97% ± 0.9) highly overlapped with the manual segmentation. On average, c8 gave a close representation of the actual volume seen in the manual segmentation, while c6 tended to underestimate and c10 to overestimate the endolymphatic space volume methodically. Note that the grade of ELH correlated significantly with the endolymphatic volume of both the manual segmentation method (two-sided, *r*(18) = 0.475, *p* = 0.034) and with VOLT cutoff variations c6 (two-sided, *r*(18) = 0.553, *p* = 0.011)–c8 (two-sided, *r*(18) = 0.566, *p* = 0.009)–c10 (two-sided, *r*(18) = 0.569, *p* = 0.009). Receiver operating characteristics (ROC) analysis showed the grade of the ELH to be a good classifier for the computed volume of the ELS (fitted ROC area: 0.9). Table 2 shows an overview of the performance and accuracy results of each segmentation method. Figure 3 gives an ear-specific overview of each validation parameter.

## Discussion

An open-source tool for automatic volumetric segmentation of the endolymphatic space (ELS) for endolymphatic hydrops (ELH) detection in intravenous, delayed, gadolinium-enhanced magnetic resonance imaging of the **i**nner ear (iMRI) data was developed on a real-world data set including 210 inner ears. The core component is a novel algorithm based on Volumetric Local Thresholding (VOLT). Tool validation in two data sets, one artificial data set that provided a known ground truth and one real-world that included 20 previously unseen inner ears, resulted in a high level of performance and accuracy in comparison with the respective gold standard (Otsu’s method and manual segmentation). In the case of the artificial data set, VOLT outperformed the gold standard in higher noise levels. VOLT endolymph volume significantly correlated with the clinical grading of the ELS. VOLT operates on a pre-segmented region-of-interest (ROI) of the inner ear, which requires a series of data pre-processing steps (duration < 10 min). Data processing steps are fully automated and run without further user input in less than 60 s.

### VOLT–performance and usability

Objective and volumetric quantification is a necessary step to assess and compare ELH results between studies and hospitals. So far, the clinical gold standard assessment of the ELS is based on a semi-quantitative and subjective grading reliant on a few MR slices in the transversal plane. In addition, different ELH classifications are being used in parallel [38, 39, 49, 50]. While manual volumetric segmentation is the gold standard for volumetric quantification, it is highly subjective and dependent on the rater’s experience and knowledge, not to mention time-consuming. VOLT allows objective, easily reproducible, and reliable stand-alone volumetric ELH quantification and grading, which closely matches manual segmentation, highly correlates with clinical ELH grading, and performs particularly well in data with a low signal-to-noise ratio.

The main advantage of VOLT is its local thresholding algorithm, which enables more flexible and stable results in comparison to global thresholding algorithms (such as Otsu’s method). Inhomogeneous image intensities and local brightness variations are adequately compensated for [48, 51]. The robustness and flexibility of VOLT to image artifacts can be further increased using different radius sizes. Importantly, results in Fig. 1 demonstrate that VOLT does not yield perfect segmentation in the absence of noise (not probable in real-world data), but instead performs more favorably and more stably in the presence of increased noise (very probable in real-world data).

Current ELS MR volumetric assessment approaches remain few and involve a manual or semi-automatic segmentation [6, 18, 50, 52]. Already a considerable improvement, these approaches require lengthy user interaction that is not suitable for use in more extensive group studies or clinical routine. Also, the software used tends to be attached to expensive software, and the uncompiled source is not available for public review.

VOLT runs smoothly and does not require especially powerful hardware or closed-source software. As an orientation, pre-processing steps of one data set can be performed in less than ten minutes by an experienced user on a standard consumer laptop. Data processing steps are fully automated and run without further user input in less than 60 s. Volumetric local thresholds can be adapted to the signal-to-noise ratio (SNR) of different data sets. Output files include 3D volumetric quantification of TLS and ELS in mm^3^ and a 3D visualization of the inner ear. The endolymphatic volumes conformed to those previously reported [53, 54].

### *VOLT flexibility*–*deep learning is beneficial but not a requirement*

Inner ear segmentation is a prerequisite step for VOLT-based ELH segmentation and is currently performed via a novel CNN-based deep learning approach [43], which is deployed as a module in 3D-Slicer [40]. This CNN was trained in-house at our department, on a separate iMRI data set obtained on the same MRI scanner and with the same imaging sequence parameters as our study. As such, this method was a natural choice for inner ear ROI segmentation in our data set, especially because segmentations were not only highly accurate but also obtainable in comparably fast execution time (< 5 s). A downside is that this network likely has difficulties in generalizing to data from other scanners or imaging sequence settings, e.g., from other clinics. Therefore, we do not assume a TOMAAT/V-Net segmentation as a fixed component of the current ELH segmentation pipeline. The inner ear ROI can also be obtained by other segmentation approaches, most prominently using atlas-based registration. Recently, two in-vivo MRI atlases and templates were proposed, one offering a probabilistic segmentation of the inner ear’s bony labyrinth [46], the other offering a high-resolution multivariate template for T1-, T2- and CISS-weighted MRI imaging [41]. Both atlases can yield accurate segmentation of the inner ear ROI while being much more generalizable to MRI data from previously unseen scanners or acquisition sites, in particular, if multivariate MRI appearances are available as in [41]. The downside of atlas-based segmentation is the high computational complexity of deformable atlas registration algorithms that align the atlas to the target volume. With carefully tuned parametrizations, such algorithms can achieve highly accurate segmentation, but segmentations can take 10 min to 2 h per volume [47], compared to < 5 s computation time for deep neural nets such as [43]. Overall, we, therefore, recommend the usage of deep neural nets for inner ear segmentation predictions; however, the correct way to generalize the network to new sites, e.g., via transfer learning, remains to be established in future work.

### Methodical limitations

There are methodical limitations in the current study that need to be considered in the interpretation of the data. First, the performance of VOLT is highly dependent on the segmentation of the inner ear. An inner ear mask that includes parts of the dark background voxels surrounding the inner ear structures would lead to a false-positive attribution to the ELS. VOLT’s high performance and accuracy values are probably in part attributable to the novel CNN-based deep learning approach. Second, VOLT does not include any anatomical knowledge. In the best case, this means that the algorithm is entirely unbiased, i.e., not influenced by any prior morphological assumptions. The downside is a lack of exclusion of apparent errors that would be noticed by the human examiner.

An example would be segmentation errors that included surrounding structures into the ROI. A human examiner would know not to expect endolymph in the outermost tips of the cochlea or vestibulum. However, an algorithm does not. This is one reason VOLT is designed unusually strict in margin areas. Finally, VOLT (or any ELS segmentation method) is by nature highly dependent upon the resolution and contrast of the MRI raw data to be able to distinguish between endolymphatic and perilymphatic space.

### Conclusion

We propose a novel pipeline for the automatic segmentation of endolymphatic hydrops in inner ear MRI. The core component is a novel algorithm based on Volumetric Local Thresholding (VOLT). Tool validation on artificial and real-world data resulted in a high level of performance and accuracy, in particular in low signal-to-noise ratio. ELS volume significantly correlated (*p* < 0.01) with the clinical grading of the ELS. A generic version of our three-dimensional thresholding algorithm has been made available to the scientific community via GitHub as an ImageJ-Plugin (https://github.com/j-gerb/3d-thresholding/tree/master).

## Abbreviations

3D: Three-dimensional
Bl: Blurriness
BPPV: Benign paroxysmal positional vertigo
BVP: Bilateral vestibulopathy
CISS: Constructive interference in steady-state
DS: Dice score
DSGZ: Interdisciplinary German Center for Vertigo and Balance Disorders
EF: Endolymphatic fluid
ELH: Endolymphatic hydrops
ELS: Endolymphatic space
FLAIR: Fluid-attenuated inversion recovery
GRAPPA: Generalized auto-calibrating partially parallel acquisition
iMRI: Delayed intravenous gadolinium-enhanced magnetic resonance imaging of the inner ear
iv: Intravenous
L: Left
R: Right
MD: Menière’s disease
MRI: Magnetic resonance imaging
NEF: Non–endolymphatic fluid
ROC: Receiver operating characteristics
Sc: Scatter
SD: Standard deviation
SVV: Subjective visual vertical
TLS: Total fluid space
U: Unclear
V_E_: Volume of the endolymphatic space
V_T_: Volume of the total fluid space
vHIT: Videooculography during the head-impulse test
VM: Vestibular migraine
VN: Vestibular neuritis
VP: Vestibular paroxysmia
